# Adrenergic receptor β_3_ is involved in the memory consolidation process in mice

**DOI:** 10.1590/1414-431X20187564

**Published:** 2018-08-06

**Authors:** P. Souza-Braga, F.B. Lorena, B.P.P. Nascimento, C.P. Marcelino, T.T. Ravache, E. Ricci, M.M. Bernardi, M.O. Ribeiro

**Affiliations:** 1 Programa de Pós-Graduação em Distúrbios do Desenvolvimento, Centro de Ciências Biológicas e da Saúde, Universidade Presbiteriana Mackenzie, São Paulo, SP, Brasil; 2 Programa de Pós-Graduação em Medicina Translacional, Departamento de Medicina, Escola Paulista de Medicina, Universidade Federal de São Paulo, São Paulo, SP, Brasil; 3 Programa de Pós-Graduação em Patologia Ambiental e Experimental, Universidade Paulista, São Paulo, SP, Brasil

**Keywords:** Adrenergic receptors, Memory, GLUT3, β_3_-AR

## Abstract

Attention and emotion have a positive impact on memory formation, which is related to the activation of the noradrenergic system in the brain. The hippocampus and amygdala are fundamental structures in memory acquisition, which is modulated by noradrenaline through the noradrenergic receptors. Pharmacological studies suggest that memory acquisition depends on the action of both the β_3_ (β_3_-AR) and β_2_ (β_2_-AR) receptor subtypes. However, the use of animal models with specific knockout for the β_3_-AR receptor only (β_3_-ARKO) allows researchers to more accurately assess its role in memory formation processes. In the present study, we evaluated short- and long-term memory acquisition capacity in β_3_-ARKO mice and wild-type mice at approximately 60 days of age. The animals were submitted to the open field test, the elevated plus maze, object recognition, and social preference. The results showed that the absence of the β_3_-AR receptor caused no impairment in locomotion and did not cause anxious behavior, but it caused significant impairment of short- and long-term memory compared to wild-type animals. We also evaluated the expression of genes involved in memory consolidation. The mRNA levels for GLUT3, a glucose transporter expressed in the central nervous system, were significantly reduced in the amygdala, but not in the hippocampus of the β_3_-ARKO animals. Our results showed that β_3_-AR was involved in the process of acquisition of declarative memory, and its action may be due to the facilitation of glucose absorption in the amygdala.

## Introduction

Attention and emotion have a positive impact on memory formation, which is related to the activation of the noradrenergic system in the brain (1,2). Norepinephrine (NE), the neurotransmitter synthesized and released by the locus coeruleus in the central nervous system (CNS), binds to the noradrenergic G-protein-coupled receptors α1, α2, b1, b2, and b3 that activate distinct pathways depending on the receptor subtype (3). The hippocampus and amygdala are critical for memory formation and consolidation, a process that is modulated by the noradrenergic system (4). Indeed, the hippocampus and amygdala express β-adrenoceptor subtypes and receive noradrenergic projections from the locus coeruleus that critically regulate behavioral memory in rodents (4,5). β-adrenoceptors (β-AR) are key for the processing of memories (6). The pharmacological blockage of β-AR with propranolol has been shown to impair the ability of rats and humans to form and consolidate long-term memory (7,8). This effect can be explained by the fact that the amygdala and hippocampus are activated during the process of acquisition and retrieval of emotional memories. Also, β-AR are expressed in both structures and propranolol blocks the increase in activity of the amygdala and hippocampus in humans after an emotional stimulus (9).

Several mechanisms have been proposed to explain the role of norepinephrine in long-term memory in mammals. Long-term potentiation (LTP), a proposed mechanism behind memory and learning, is modulated by NE acting on β-AR to enhance LTP and boost memory endurance (3,10,11). Additionally, β-AR activation increases the expression of the a-amino-3-hydroxy-5-methyl-4-isoxazoleproprionic acid (AMPA) receptor subunit GluA1, one of the four subunits of the AMPA receptor for glutamate (12). NE also increases memory consolidation through an increase in glucose availability in the hippocampal neurons as shown by studies performed in chickens with zinterol and CL316243, a β_2_-AR agonist and a β_3_-AR agonist, respectively (13). This occurs through the increased breakdown of glycogen by phosphorylase glycogen brain (PYGB) activation in response to β_2_-AR activation, while β_3_-AR mediates the glucose uptake by the glucose transporter type 3 (GLUT3). These studies suggested that β_3_-AR increases glucose uptake in an early stage, whereas the β_2_-AR increases glucose availability at a later stage, suggesting a synergic effect of both adrenoceptors in regulating short- and long-term memory, respectively.

To better understand the mechanisms underlying the role of the β_3_-AR in learning, the memory in knockout mice for this receptor (β_3_-ARKO) was evaluated. Thus, we employed two behavioral models related to declarative memory, novel object recognition (14), and social preference and discrimination tests (15). In addition, emotionality and motor aspects in these mice were investigated by the open field and elevated plus maze tasks, since motor impairments and anxiety could interfere with the learning performance in learning models. Our study shows for the first time that β_3_-AR plays a role in short- and long-term memory and suggests that it could be related to GLUT3 expression.

## Material and Methods

### Animals

β_3_-ARKO mice, generated by removing the 306 bp genomic fragment containing the sequences encoding the third through the fifth transmembrane domains of the β_3_-AR and replacing it with a neomycin selection cassette as described by Susulic et al. (16), were obtained from Jackson Laboratory (Bar Harbor, USA). All animals were genotyped to confirm their status as homozygous knockout or wild-type (WT). Homozygous β_3_-ARKO mice are unresponsive to β_3_-AR agonist treatment. All animals were maintained on an FVB background. Approximately 60-day-old male β_3_-ARKO mice and WT controls were used following the animal protocol approved by the Institutional Committee on Animal Research at the Center of Biological Sciences and Health, Mackenzie Presbyterian University. Each experiment was repeated two or three times on different sets of animals (n=8). Mice were housed in groups under standard conditions at 26°C, 55–60% humidity, and a 12-h light/dark cycle with *ad libitum* access to standard food (Nuvilab, Brazil) and water.

### Behavioral Testing

#### Open Field

The open field test (OFT) was used for assessment of locomotor and exploratory activity of the mice (17). The apparatus was located in a 1.8 × 4.6 m test room and lit by a 15-lux lamp for background lighting; the apparatus was washed with a 5% alcohol–water solution before placement of the animals to obviate bias caused by odor cues left by previous animals. The animals were tested in a square acrylic arena (72 × 72 cm) surrounded by a 30-cm wall. The floor of the arena was divided into sixteen 18 × 18 cm squares, and mice were placed into the center of the arena and observed for 5 min; all tests were recorded by video camera for later analysis. The following parameters were measured: 1) total locomotion (total number of lines crossed with four paws); 2) grooming (number of times cleaning the body with paws, grooming the body and pubis with the mouth, and face washing, and 3) rearing (number of times the rodents stood on their hind legs).

#### Elevated Plus Maze (EPM)

The EPM was made of plastic covered plywood and consisted of two opposed open arms (30 × 5 cm) and two opposed enclosed arms (30 × 5 × 15 cm) connected by a central open square (5 × 5 cm) (17). The maze was elevated 38 cm above the floor and placed inside a room free from noise and disturbances, under dim laboratory light and the test was recorded by video camera for later analysis. The animals were placed into the central square of the maze facing one of the open arms and observed for 5 min. The percentage of time spent on the center and on open and closed arms (arm entry = all four paws on an arm) and number of head-dips (exploratory movement of head/shoulders over the side of the maze) were evaluated (18).

#### Novel object recognition

For assessment of novel object recognition, the animals were placed in the open field apparatus used to assess their locomotor activity so the environment would not be new to them. The objects used during testing were Lego® figures of equal size and material that differed in design. These were weighted to minimize the movement of the objects by the mice during the trials. The trials were video-recorded and later coded by an experimenter who was blind to the treatment groups. The objects to be used were first assessed to ensure that there were neither intrinsic preferences nor aversions, and that each object would be explored for similar durations upon initial exposure. Exploration was considered when the mice directed the nose to the object at a distance of no more than 2 cm and/or touching the object with the nose or mouth. Rearing up on the object was counted only if facing toward, but not away from the object (19,20). Total time spent exploring each object was recorded over a 5-min period. At the end of the first trial, the mice were returned to their home cage for 3 h. Afterwards, each mouse was returned to the same testing cage for the second trial, which contained one of the previous objects (a familiar object) and a novel object, placed in the same locations. The total time spent exploring each object was recorded over a 5-min period. At the end of the second trial, the animals were returned to their home cage for 24 h. Then, each mouse was returned to the same testing cage for the third trial, which contained the same familiar object and another novel object, placed in the same locations.

#### Social preference and discrimination

The parameters were evaluated using a non-automated 3-chamber box with three successive and identical chambers (Stoelting, Ireland) as described previously (21–23). During habituation, the mice explored the three chambers freely for 10 min from the intermediate compartment, with the two other chambers containing empty wire cages. In the second phase, to test social preference, the mouse was placed in the central box, while an unknown mouse (stranger 1) was in one of the wire cages in a random and balanced manner and the mouse was allowed to explore the three chambers for 10 min. The time spent in each of the chambers, the number of entries into each chamber, and the time spent sniffing each wire cage were recorded. In the third phase, social memory was evaluated with a new unknown mouse (stranger 2) in the remaining empty wire cage, with the mouse allowed to explore the entire arena for 6 min. The time spent with the previously investigated mouse (stranger 1) and the novel unknown mouse (stranger 2) and the same measures were recorded for the social preference evaluation.

### Gene expression analysis

#### mRNA

Total tissue RNA was extracted from the hippocampus and amygdala using the RNeasy® Plus Universal Mini Kit (Qiagen, USA), following the manufacturer's instructions. RNA was quantified with a NanoDrop spectrophotometer, and 0.5–1.0 μg total RNA was used to produce cDNA using the SuperScript® III First-Strand Synthesis SuperMix for qRT-PCR (Invitrogen, USA). RT-qPCR was performed as previously described (24). Genes of interest (sequences upon request) were measured (Step-one Applied Biosystem, USA) using QuantiTect™ SYBR® Green (Qiagen, USA). The melting curve protocol was performed to verify the specificity of the amplicon generation. Standard curves consisted of 4–5 points of serially diluted mixed experimental and control group cDNA. Cyclophilin A (CycloA) was used as a housekeeping internal control gene. The coefficient of correlation was greater than 0.99 for all curves, and the amplification efficiency ranged between 80 and 110%. Results were reported as the ratio of test mRNA to CycloA mRNA. The following genes were studied: b1 adrenergic receptor (*Adrb1*), b2 adrenergic receptor (*Adrb2*), glucose transporter 3 (*Slc2a3*), phosphorylase glycogen b (*Pygb*), brain-derived neurotrophic factor (*Bdnf*), and nerve growth factor (*Ngf*).

### Statistical analyses

All data are reported as means±SEM and were analyzed using PRISM software (GraphPad Software, USA). Student's *t*-test was used to compare the groups WT and β_3_-ARKO. P<0.05 was used to reject the null hypothesis.

## Results

### β_3_-ARKO mice exhibited impairment in short- and long-term memory

**Figure 1.**
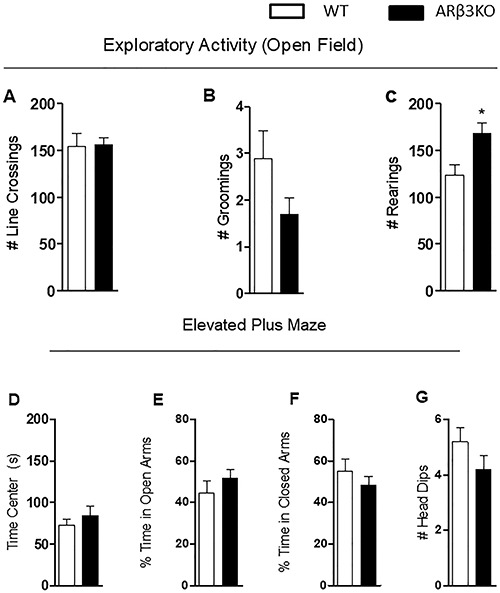
Loss of β_3_-AR does not affect locomotor activity or anxiety behavior in mice. *A*–*C*, Open field: *A*, Total number of line crossings; *B*, Total number of groomings; *C*, Total number of rearings. *D*–*G*, Elevated plus maze: *D*, Time spent in center of apparatus; *E*, Time spent in open arms; *F*, Time spent in closed arms; *G*, Total number of head-dips. *P<0.02 *vs* wild-type (WT). Data are reported as means±SEM (n=10) (Student's *t*-test).

To evaluate if the absence of this adrenoceptor would impact their ambulatory and exploratory activity, β_3_-ARKO mice were exposed to the open field under conditions of low light intensity, which sets a low emotionality context. No differences in line crossing and grooming were detected, but an increase in rearing was observed in the β_3_-ARKO mice (P<0.02 and t= 2.82) (Figure 1A, B, and C).

Next, the animals were studied in the elevated plus maze to assess anxiety (17). No difference was observed in the time spent in the center, open, or closed arms (Figure 1D, E, and F) and in the number of head-dips (Figure 1G).

**Figure 2.**
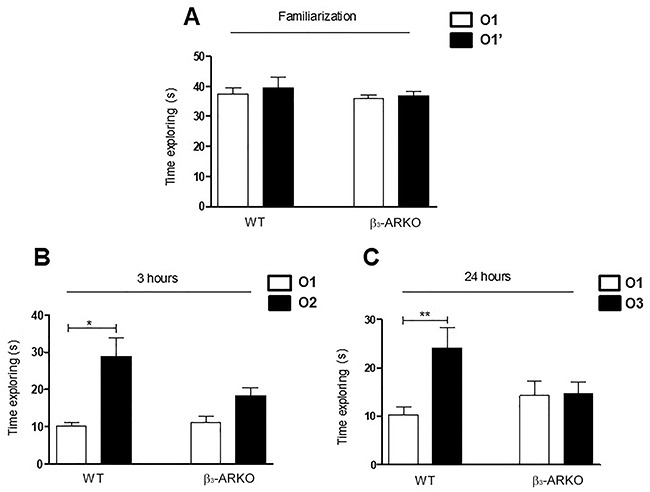
β_3_-AR is involved in memory consolidation. *A*, Wild-type (WT) and β_3_-ARKO mice spent the same amount of time with a familiar object (O1) and a similar object (O1′) during object familiarization. *B*, Three hours after object familiarization, WT mice spent significantly more time with a novel object (O2) than a familiar object (O1) during object recognition, while β_3_-ARKO mice spent an equal amount of time with both O1 and O2. *C*, Twenty-four hours after object familiarization, WT mice spent significantly more time with a novel object (O3) than a familiar object (O1) during object recognition, while β_3_-ARKO mice spent an equal amount of time with both O1 and O3. Data are reported as means±SE (n=10). *P<0.01, **P<0.05 *vs* WT (ANOVA followed by the Student Newman-Keuls test).

Animals were then tested for cognition using the novel object recognition task. In the training phase, both groups spent a similar amount of time with both objects 01 and 01' (Figure 2A). When the mice were presented with a new object (02) 3 h later, WT mice spent 3-fold more time with the new object (02) than with the familiar object (01) (P<0.001, F=8.795). However, the β_3_-ARKO mice spent an equal amount of time with the familiar object (01) as with the novel object (02) (Figure 2B). Similar results were obtained when the mice were presented 24 h later to another new object (03); WT mice spent 2.5-fold more time with the new object than the familiar object (01) (P<0.05, F=3.982) while β_3_-ARKO mice spent an equal amount of time with both (Figure 2C) (17).

**Figure 3.**
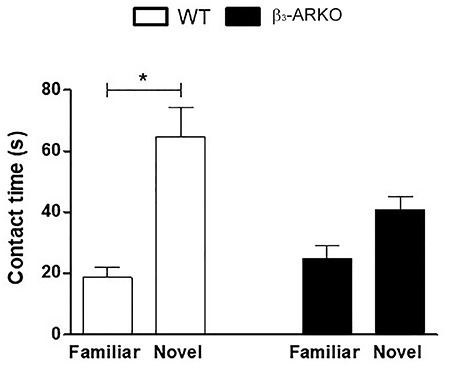
β_3_-AR is important for social discrimination. Wild-type (WT) mice showed normal preference for social novelty and spent significantly more time in the chamber with the novel mouse than in the chamber with the familiar mouse. β_3_-ARKO spent the same amount of time with the familiar mouse and with the novel mouse. Data are reported as means±SE (n=10). *P<0.001 (ANOVA followed by the Student Newman-Keuls test).

In the social discrimination test, β_3_-ARKO mice were tested regarding their ability to discriminate strangers from familiar conspecifics. In the first phase of the test, both groups explored all the compartment of the apparatus equally (data not shown). In the third phase, the mice were presented to familiar and unfamiliar conspecifics. The WT mice presented a significant preference for the newly introduced mouse compared to the familiar animal (P<0.001, F=11.86), whereas β_3_-ARKO mice did not show this discrimination (Figure 3).

### Decreased *Slc2a3* expression in the amygdala but not in the hippocampus of β_3_-ARKO mice

**Figure 4.**
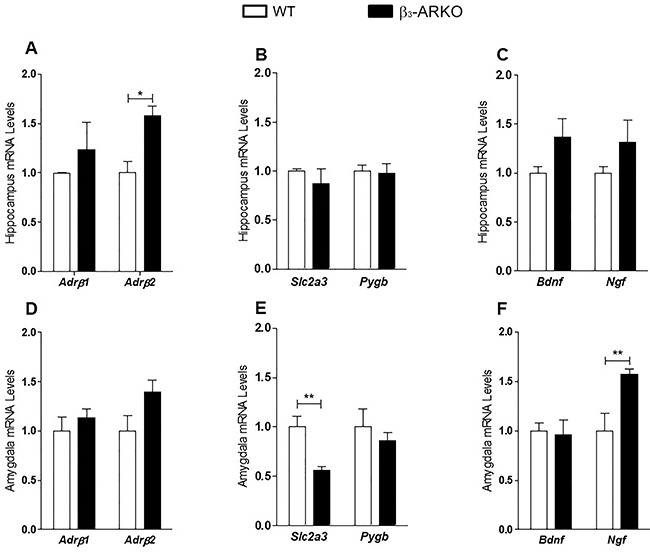
Gene expression (mRNA levels) in the hippocampus (*A–C*) and in the amygdala (*D–F*) of wild-type (WT) and β_3_-ARKO mice measured by RT-qPCR and using CycloA as internal control. Data are reported as means±SE (n=4). *P<0.001, **P<0.03 (Student's *t*-test).

We first evaluated if the lack of β_3_-AR would lead to an increase in the *Adrb1* and *Adrb2* mRNA expression as a compensatory mechanism. The mRNA levels of *Adrb1* were the same in the hippocampus and amygdala of β_3_-ARKO mice compared to WT, but there was a significant increase in mRNA levels of *Adrb2* mRNA in the hippocampus of the β_3_-ARKO mice compared to WT (P<0.01, t=3.85) (Figure 4A and D).

To understand the mechanisms underlying the lack of memory and social recognition, RT-qPCR was used to measure mRNA levels of neurotrophins and of proteins involved in glucose metabolism in the hippocampus and amygdala. No difference was found for *Slc2a3*, *Pygb*, *Bdnf*, and *Ngf* mRNA levels in the hippocampus (Figure 4A to C). However, we found a decrease in *Slc2a3* mRNA (P<0.03, t=2.902) and an increase in *Ngf* mRNA levels in the amygdala (P<0.03, t=3.033) (Figure 4E and F).

## Discussion

Our results showed for the first time that β_3_-AR is relevant for declarative memory acquisition and consolidation, since its absence in mice significantly impaired these processes despite an overexpression of *Adrb2* in the hippocampus. We also showed that this could be due to the decreased gene expression of *Slc2a3* in the amygdala. Motor impairments or anxiety-like behaviors were not involved in the lack of memory acquisition and social recognition in β_3_-ARKO mice.

Studies performed in chicks using pharmacological approaches have suggested that both the β_2_-AR and β_3_-AR receptors are important in the process of the acquisition of declarative memory (25). Memory acquisition and consolidation mediated by β-ARs involve the activation of the glucose metabolism (26). Norepinephrine leads to increased GLUT3 expression in the membrane of neurons through the activation of β_3_-AR, while activation of β_2_-AR leads to increased activity of the PYGB. The combined action of the two isoforms results in an increase in the glucose available to be used by the hippocampal neurons. Therefore, the NE effect would depend on the presence and concomitant activation of both β_2_-AR and β_3_-AR receptors and that the activation of β_2_-AR would be sufficient for the acquisition of memory during the blocking of β_3_-AR (13). However, our results contradicted these findings, as they showed that even in the presence of an increase in β_2_-AR gene expression, memory consolidation and social recognition were impaired in β_3_-ARKO mice.

We also showed that in the absence of the β_3_-AR there was a significant decrease in gene expression of *Slc2a3* in the amygdala, but not in the expression of *Pygb*. It is surprising, however, that there was impaired memory formation despite normal levels of mRNA for *Pygb*, suggesting that GLUT3 was critical to this process. Although the literature shows that the activation of PYGB is key to memory formation, our results showed that the impairment of memory formation occurred even in the presence of normal mRNA levels for this enzyme.

It has been shown that norepinephrine significantly increases in the frontal cortex and hypothalamus of rats exposed to new settings and an unfamiliar rat, mediating long-term memory formation (27), suggesting that NE is important in mediating the response to novel stimulus. The increase in NE levels leads to an increase in excitability of neurons from hippocampus via activation of β-AR (28–30).

β_3_-AR are highly expressed in the subgranular zone of the dentate gyrus (DG) during development (31). Also, there is a very dense innervation to DG originating from LC with consequent higher levels of NA in DG compared with CA1 and CA3 regions (32). Taken together, these data could explain why the lack of β_3_-AR in β_3_-ARKO mice results in a significant impairment in memory consolidation.

In our model, memory formation impairment was not related to BDNF and NGF, classically known neurotrophin mediators of synaptic plasticity (33,34). In fact, there was an increased expression of *Ngf* in the amygdala of the β_3_-ARKO mice, which was not able to restore the memory acquisition capacity.

Although social recognition, a process that involves the amygdala (35,36), was significantly impaired in the β_3_-ARKO mice, the total contact time between β_3_-ARKO and known and unknown animals was equal to that of the WT mice, suggesting that there was no impairment to social interaction (37,38).

We observed that β_3_-ARKO animals did not show anxious behavior. These results are surprising considering that the *locus coeruleus* sends projections to the amygdala, the structure of the limbic system responsible for the recognition and mediation of fear and anxiety. Moreover, the use of selective β_3_-AR agonists induces a significant anxiolytic-like effect when administered orally to mice (39,40).

Taken together, our data showed that β_3_-AR receptors were involved in declarative memory consolidation.
